# Day-3-embryo fragmentation is associated with singleton birth weight following fresh single blastocyst transfer: A retrospective study

**DOI:** 10.3389/fendo.2022.919283

**Published:** 2022-09-23

**Authors:** Jiali Cai, Lanlan Liu, Jinghua Chen, Zhenfang Liu, Xiaoming Jiang, Haixiao Chen, Jianzhi Ren

**Affiliations:** ^1^Reproductive Medicine Centre, Affiliated Chenggong Hospital of Xiamen University, Xiamen, China; ^2^School of Medicine, Xiamen University, Xiamen, China

**Keywords:** fragmentation, birth weight, large for gestational age, blastocyst, inner cell mass

## Abstract

**Background:**

Previous studies have arguably associated poor embryo morphology with low birth weight in singletons following single embryo transfer. However, the association between birth weight and specific morphological features in the cleavage stage remains less known. The purpose of the study was to investigate whether morphological features of embryos at the cleavage stage affect birth weight following blastocyst transfer.

**Methods:**

The single-center retrospective cohort study included 4,226 singletons derived from fresh single cleavage-stage embryo transfer (ET; n = 1,185), fresh single blastocyst transfer (BT; n = 787), or frozen-thawed single blastocyst transfer (FBT; n = 2,254) between 2016 and 2019. Morphological parameters including early cleavage, day-3 fragmentation, symmetry, blastomere number, and blastocyst morphology were associated with neonatal birth weight and birth weight z-score in multivariate regression models. The models were adjusted for maternal age, body mass index (BMI), parity, peak estradiol level, endometrial thickness, insemination protocol, female etiologies, order of transfer, mode of delivery, and year of treatment.

**Results:**

Adjusted for confounders, day-3 fragmentation was the only morphological feature associated with birth weight and birth weight z-score, while early cleavage, symmetry, blastomere number, and blastocyst morphology were not. Day-3 fragmentation increased the birth weight in both the ET (115.4 g, 95% CI: 26.6–204.2) and BT groups (168.8 g, 95% CI: 48.8–288.8) but not in the FBT group (7.47 g, 95% CI: -46.4 to 61.3). The associations between birth weight and these morphological parameters were confirmed through birth weight z-score analyses. The adjusted odds of large for gestational age (LGA) and high birth weight were also significantly greater in singletons following the transfer of fragmented embryos in the BT group [odds ratio (OR) 3, 95% CI: 1.2–7.51 and OR 3.65, 95% CI: 1.33–10, respectively]. The presence of fragmentation at the cleavage stage also affected the association between the blastocyst morphology and birth weight. Inner cell mass grades were negatively associated with birth weight in blastocysts with day-3 fragmentation but not in blastocysts without.

**Conclusions:**

The birth weight following blastocyst transfer was found to be positively associated with fragmentation at the cleavage stage. The data did not support the argument that transferring a poor-looking embryo may increase the risks of low birth weight. However, concerns for LGA infants remain.

## Introduction

Despite the rapid evolution of embryo assessment technologies that has occurred within the past decade, the conventional embryo morphology assessment based on microscopic observation remains a routine procedure for many embryo laboratories and continues to play a key role in quality control systems (1). Morphological features observed within a fixed period post-insemination, such as the degree of fragmentation, rate of cell division, and size of blastomeres, as well as the morphologies of the trophectoderm (TE) and inner cell mass (ICM), have been proposed as indicators of embryo quality and developmental potential (2). Embryos with one or more unfavorable morphological features are believed to be of poorer quality and less likely to result in a live birth.

Despite this lower chance, achieving a live birth is still possible following the transferring of embryos with poor morphology. This raises the question of whether children derived from poor-looking embryos differ from those with “standard” morphology at their early stage of life. Several investigators have evaluated the association between embryo quality according to morphology and neonatal outcomes, showing that transferring poor-quality embryos at either the cleavage or blastocyst stage is associated with low birth weight in offspring (3, 4). It suggests a potential link between embryo morphology and fetal development. However, their results conflict with those of other studies (5–7) demonstrating a lack of association between embryo morphology and birth weight; furthermore, they are hampered by the subjectivity of the criteria. Other authors have associated birth weight with specific morphological parameters, such as day-3 cell number, advanced ICM grades, and TE morphology (8–11). These data suggest that specific parameters rather than overall embryo grading might have an impact on birth weight. However, whether or how these morphological parameters interact or work in combination with each other remains unclear.

Recent data suggest that unfavorable morphological features at the cleavage stage may impair implantation and live birth following blastocyst transfer, even when high-quality blastocysts were transferred (12–14). Such studies highlight the need to combine evaluations of the cleavage stage and blastocyst morphology to assess the developmental potential of a blastocyst. We hypothesize that the combination of cleavage stage and blastocyst morphology may also be linked to birth weight variation following *in vitro* fertilization (IVF), even though the morphological grades of the transferred blastocysts were similar. The present study aimed to evaluate the association between day-3 morphological features and birth weight in both the cleavage stage and blastocyst transfer, in addition to examining whether day-3 morphology modifies the association between birth weight and blastocyst morphology.

## Methods

### Study subjects

Institutional review board approval for this retrospective study was obtained from the ethical committee of the Xiamen University Affiliated Chenggong Hospital.

Data of all patients who underwent single embryo transfer in either fresh or frozen-thawed cycles in the affiliated Chenggong Hospital of Xiamen University between January 2016 and December 2019 were accessed for potential inclusion. Only cycles resulting in singleton live birth were included. The exclusion criteria were a vanishing twin, very preterm birth (gestational week <26), patients reporting maternal gestational hypertension or diabetes, uterine abnormalities, and unknown perinatal outcomes due to incomplete records. To minimize the maternal confounding, we also excluded maternal metabolism-related conditions, including Polycystic ovary syndrome (PCOS) and glucose intolerance. Female smokers were excluded from the study rather than being considered confounders because of the small number of events (n = 28). The present study did not analyze the embryos with early compaction on day 3 because our grading system did not record the cell number or fragmentation if early compaction had occurred. A previous study has suggested that the effects of early compaction may be modified by other morphological parameters (15).

### Laboratory procedures and embryo assessment

Patients received ovarian stimulation as previously described (16). Briefly, agonist or antagonist stimulation protocols were used, and the starting dose of gonadotropin was adjusted according to the patient’s age, antral follicle count (AFC), body mass index (BMI), and follicular growth response. All patients received 1–3 ampules (75–225 IU) of gonadotropin per day. Following ovum pick-up (OPU), oocytes were inseminated using either conventional IVF or intracytoplasmic sperm injection (ICSI), with the fertilization results checked 18–20 h following insemination. Embryos and blastocysts were all cultured in single droplets following insemination. All of the oocytes and embryos were cultured in Cook series media (KSIFM, KSICM, or KSIBM, Cook Medical, Bloomington, IN) with oil overlay (OVOIL, Vitrolife, Göteborg, Sweden) in traditional incubators (C200, Labotect, Göttingen, Germany) at 37°C, 6% CO_2_, and 5% O_2_.

Morphological evaluations were performed on days 1, 3, and 5 of the culture. In this study, all of the morphological evaluations at the cleavage and blastocyst stages were conducted by two of the authors (XJ and ZL). Each has over a decade of experience as an embryologist.

On day 1, the occurrence of early cleavage was observed. On day 3, the embryo quality was assessed through a combination of cell number, blastomere size, and degree of fragmentation. Grade I embryos (good quality) were defined as eight-cell embryos with evenly sized blastomeres and no more than 5% fragmentation. Grade II embryos (fair quality) were deemed 7–10-cell embryos with minor defects in blastomere size or moderate fragmentation (10%–15%). Grade III embryos (poor quality) were embryos with any two of the following defects: cell number <7 or >10, abnormally sized blastomeres, and a fragmentation rate of ≥10%. Embryos with severe fragmentation (≥50%) or those with a combination of more than two major defects were not considered for transfer. Day-3 fragmentation here was defined as small anuclear extracellular cytoplasmic structures. Due to the subjectivity of the fragmentation evaluation, representative images for day-3 embryos with different fragmentation degrees in our clinic are shown (**Supplementary Figure S1**).

The Gardner grading system (1) was utilized for the blastocyst assessment. The degree of expansion (1: the blastocoel is <50% volume of the embryo; 2: the blastocoel is ≥50% volume of the embryo; 3: the blastocoel fills the whole embryo; 4: the blastocoel volume is increased with zona thinning; 5: the TE partially escapes from the zone; 6: the blastocyst completely hatches from the zona), the morphology of the ICM (A: many cells, tightly packed; B: fewer cells, loosely packed; C: very few cells), and the morphology of the TE (A: a cohesive epithelium with many cells, typically >12 cells at the equator of the blastocyst; B: a loose epithelium with fewer cells, typically 7–12 cells at the equator of the blastocyst; C: very few large irregularly sized cells, typically <7 cells at the equator of the blastocyst) were evaluated accordingly. Blastocysts with poor morphological scores (≤CC) or low expansion grades (grades 1–2) were not considered for cryopreservation or transfer. To facilitate the comparison of the various results, quality categories similar to those employed in a previous study (4) were used in the present study: top quality: AA; good quality: AB and BA; average quality: AC, CA, and BB; and poor quality: BC and CB.

For the cryopreservation of the embryo, a vitrification protocol was used, which employed 15% dimethyl sulfoxide, 15% ethylene glycol, and 0.6 M sucrose as cryoprotectants. The blastocoelic volume was reduced before cryopreservation using a laser system (RI Saturn, Falmouth, UK).

Embryo transfers were performed using a Cook catheter (K-JETS-7019-SIVF, Cook, IN, USA) under the guidance of abdominal ultrasonography. Assisted hatching was not performed during the period of the study. Luteal support continued until 10 weeks of pregnancy.

### Statistics

The main outcomes measured were absolute birth weight and birth weight z-score adjusted for gestational age and gender. The birth weight z-score was calculated as the weight of the individual child minus the median weight of a reference population of children born at the same gestational age and of the same gender divided by the standard deviation from the same reference population. For the calculation, we used a birth weight reference for the Chinese population generated by the national population-based Birth Defects Surveillance System (17). The small-for-gestational-age (SGA) and large-for-gestational-age (LGA) calculations used the same reference. LGA was defined as newborns with a birth weight >90th percentile for that gestational age and gender. SGA was defined as newborns with a birth weight <10th percentile for that gestational age and gender.

The association between the embryo morphological parameters and birth weight outcomes was analyzed using generalized linear models (GLMs). The models were adjusted for a set of covariates that may affect the birth weight outcomes following embryo transfer; these included maternal age, BMI, parity, peak estradiol level, and endometrial thickness. Also incorporated were potential confounders, including the insemination protocol (IVF or ICSI), female etiologies (tubal factor, endometriosis), order of transfer (1 or >1), mode of delivery (vaginal or Cesarean), and year of treatment. To analyze the absolute birth weight, the models were also adjusted for gestational age and gender. The same set of covariates was also used in the multivariate analyses for LGA, SGA, high birth weight (HBW; >4,000 g) and low birth weight (LBW; <2,500 g).

The morphological parameters evaluated were early cleavage on day 1 (yes or no), fragmentation (<10% or ≥10%), cell number (<8 cells, 8 cells, or >8 cells), and symmetry (even or uneven) on day 3, as well as ICM/TE (grade A, B, or C) morphology on day 5. We used two methods to evaluate the combinational effects of the cleavage stage and blastocyst morphology on birth weight. First, the morphological parameters were assumed to be independent and to serve as confounders to each other in the multivariate models. Second, interactions between morphological parameters were introduced into the multivariate models. To simplify the interpretation of the data, only two-way interactions were considered. The postulated interaction patterns were first checked in a main-effect-only model and then confirmed in a multivariate model.

All of the calculations were performed using SPSS (version 19; IBM).

## Results

A total of 5,228 singleton live births following single embryo transfer were reviewed for potential inclusion. After excluding incomplete records (n = 20), very preterm births (n = 20), vanishing twin (n = 27), reported gestational diabetes (n = 26) and hypertension (n = 59) cases, uterine abnormalities (n = 28), glucose intolerance or diagnosed diabetes (n = 21), PCOS (n = 470), female smokers (n = 28), and early compaction (n = 176), 4,353 singletons were included. Of these, 1,185 were following fresh cleavage-stage transfer, 127 were following frozen-thawed cleavage-stage transfer, 787 were following fresh blastocyst transfer, and 2,254 were following frozen-thawed blastocyst transfer. Due to the limited sample size, we did not further analyze singletons following frozen-thawed cleavage-stage transfer; the characteristics, outcomes, and multivariate analyses for these patients are detailed in the supplementary material (**Supplementary Tables S1**, **S2**).

For singletons following fresh single cleavage-stage embryo transfer (ET), fresh single blastocyst transfer (BT), and frozen-thawed single blastocyst transfer (FBT), the patient characteristics, embryo parameter distribution, and neonatal outcomes are provided in **Table 1**. The median [interquartile range (IQR)] of the maternal age and the BMI of the overall population were 30 (28–33) years and 20.9 (19.4–22.5) kg/m^2^, respectively. The median birth weight, birth weight z-score, and gestational age of the singletons were 3,250 (3,000–3,500) g, -0.11 (-0.74 to 0.55), and 39 (38–40) weeks, respectively.

**Table 1:** Patients’ characteristics and neonatal outcomes.

Variable			Fresh cleavage	Fresh blastocyst	Frozen blastocyst
N			1,185	787	2,254
Maternal age, years			31 [28-34]	31 [28-33]	30 [27-32]
Maternal BMI, kg/cm^2^			21.2 [19.6-22.7]	21.3 [19.7-22.69]	20.7 [19.1-22.3]
Maternal etiology		Tubal	137 (11.56)	88 (11.18)	204 (9.05)
		Endometriosis	760 (64.14)	524 (66.58)	1,495 (66.33)
Insemination		IVF	858 (72.41)	587 (74.59)	1,634 (72.49)
		ICSI	327 (27.59)	200 (25.41)	620 (27.51)
ET order		1	193 (16.29)	222 (28.21)	320 (14.20)
		>1	992 (83.71)	565 (71.79)	1,934 (85.80)
Parity		0	827 (69.79)	568 (72.17)	1,922 (85.27)
		≥1	358 (30.21)	219 (27.83)	332 (14.73)
Endometrial thickness, mm			10.8 [9.4-12.6]	11 [9.4-12.7]	8.8 [7.8-10.1]
Peak estradiol level, pg/L			2,196.5 [1,128.25-3,788.75]	3,597 [2,305-4,552]	291 [208-398]
Stimulation protocol		Agonist	952 (80.34)	731 (92.88)	2,093 (92.86)
		Non-agonist	233 (19.66)	56 (7.12)	158 (7.14)
Year of treatment		2016	117 (9.87)	92 (11.69)	158 (7.01)
		2017	336 (28.35)	100 (12.71)	432 (19.17
		2018	320 (27.00)	166 (21.09)	566 (25.11)
		2019	412 (34.77)	429 (54.51)	1,098 (48.71)
**Embryo features**					
Early cleavage			834 (70.38)	577 (73.32)	1,477 (65.53)
Day-3 fragmentation ≥10%			85 (7.17)	48 (6.10)	241 (10.69)
Day-3 cleavage		8 cells	937 (79.07)	532 (67.60)	1,200 (53.24)
		<8 cells	135 (11.39)	88 (11.18)	501 (22.23)
		>8 cells	113 (9.54)	167 (21.22)	551 (24.45)
Day-3 asymmetry			160 (13.50)	120 (15.25)	493 (21.87)
Blastocyst ICM		A	–	126 (16.01)	488 (21.65)
		B	–	649 (82.47)	1,727 (76.62)
		C	–	12 (1.52)	39 (1.73)
Blastocyst TE		A	–	292 (37.10)	864 (38.33)
		B	–	479 (60.86)	1,320 (58.56)
		C	–	16 (2.03)	70 (3.11)
Cleavage score		Grade III	91 (7.68)	93 (11.82)	476 (21.12)
		Grade II	983 (82.95)	661 (83.99)	1,696 (75.24)
		Grade I	111 (9.37)	33 (4.19)	80 (3.55)
Blastocyst score		Top		86 (10.93)	327 (14.51)
		Good		246 (31.26)	697 (30.92)
		Fair		427 (54.26)	1,122 (49.78)
		Poor		28 (3.56)	108 (4.79)
**Neonatal outcomes**					
Mode of delivery		Vaginal	493 (41.60)	339 (43.07)	889 (39.44)
		Cesarean	692 (58.40)	448 (56.93)	1365 (60.56
Offspring gender		Female	557 (47)	346 (44.96)	965 (42.81)
		Male	628 (53)	441 (56.04)	1,289 (57.19)
Birth weight, g			3,200 [2,900-3,500]	3,200 [2,920-3,500]	3,250 [3,000-3,550]
Birth weight z-score			-0.22 [-0.8 to 0.42]	-0.22 [-0.8 to 0.54]	-0.06 [-0.63 to 0.65]
Gestational age, weeks			39 [38-40]	39 [38-40]	39 [38-40]
Preterm birth			59 (4.98)	43 (5.46)	120 (5.32)
LBW, <2,500 g			69 (5.82)	54 (6.86)	129 (5.72)
HBW, >4,000 g			65 (5.49)	42 (5.34)	139 (6.17)
LGA			105 (8.86)	64 (8.13)	216 (9.58)
SGA			148 (12.49)	99 (12.58)	196 (8.70)

ET, embryo transfer; ICM, inner cell mass; TE, trophectoderm; LBW, low birth weight; HBW, high birth weight; LGA, large for gestational age; SGA, small for gestational age.

The associations between the birth weight z-score and each morphological feature of the transferred embryos are provided in **Table 2**. Among the morphological features evaluated in the present study, embryo fragmentation was the only one to be associated with the birth weight z-score of singletons following transfer. In both the ET cycles and BT cycles, the singleton birth weight z-score following transfer of the fragmented embryos (≥10%) on day 3 was significantly higher than the score after transfer of embryos with no or little fragmentation (0.17 vs. -0.15 for cleavage, 0.22 vs. -0.13 for blastocyst). In the FBT cycles, however, fragmentation on day 3 appeared to have no impact on the birth weight z-score (0.06 vs. 0.02). Adjusted for the aforementioned covariates, the association between day-3 fragmentation and birth weight z-score remained significant in ET and BT cycles (**Table 2**).

**Table 2:** Association between embryo morphological features and birth weight z-score.

Variable	category	Fresh cleavage transfer				Fresh blastocyst transfer				Frozen blastocyst transfer			
		unadjusted		adjusted		unadjusted		adjusted		unadjusted		adjusted	
		coefficient(95% CI)	p	coefficient(95% CI)	p	coefficient(95% CI)	p	coefficient(95% CI)	p	coefficient(95% CI)	p	coefficient(95% CI)	p
Early cleavage	yes	-0.01 (-0.14 to 0.13)	0.91	-0.01 (-0.14 to 0.12)	0.91	0.12 (-0.05 to 0.29)	0.16	0.11 (-0.07 to 0.28)	0.23	0.04 (-0.05 to 0.13)	0.38	0.05 (-0.04 to 0.14)	0.30
	no	Ref	–	Ref	–	Ref	–	Ref	–	Ref	–	Ref	–
Day-3 Fragmentation	≥10%	**0.33 (0.09 to 0.56)**	**0.01**	**0.27 (0.03 to 0.5)**	**0.02**	**0.35 (0.04 to 0.66)**	**0.03**	**0.42 (0.11 to 0.74)**	**0.01**	0.04 (-0.1 to 0.17)	0.61	0.02 (-0.12 to 0.16)	0.74
	<10%	Ref	–	Ref	–	Ref	–	Ref	–	Ref	–	Ref	–
Cell number	>8 cells	0.18 (-0.03 to 0.38)	0.10	0.15 (-0.06 to 0.36)	0.16	0 (-0.19 to 0.19)	0.99	-0.04 (-0.23 to 0.16)	0.71	0.09 (-0.02 to 0.19)	0.10	0.06 (-0.05 to 0.16)	0.27
	<8 cells	0.08 (-0.11 to 0.27)	0.40	-0.05 (-0.25 to 0.15)	0.599	0.04 (-0.2 to 0.28)	0.744	0.12 (-0.12 to 0.36)	0.324	0.11 (0 to 0.21)	0.054	0.08 (-0.03 to 0.19)	0.162
	8 cells	Ref	–	Ref	–	Ref	–	Ref	–	Ref	–	Ref	–
Symmetry	uneven	-0.01 (-0.18 to 0.17)	0.95	-0.09 (-0.27 to 0.09)	0.33	0.02 (-0.19 to 0.23)	0.84	0.04 (-0.18 to 0.26)	0.71	0.09 (-0.01 to 0.19)	0.08	0.09 (-0.01 to 0.19)	0.09
	even	Ref	–	Ref	–	Ref	–	Ref	–	Ref	–	Ref	–
Cleavage score	Grade I	-0.15 (-0.44 to 0.15)	0.32	0 (-0.3 to 0.3)	0.99	-0.13 (-0.56 to 0.29)	0.54	-0.12 (-0.54 to 0.3)	0.59	-0.17 (-0.41 to 0.07)	0.17	-0.12 (-0.36 to 0.12)	0.33
	Grade II	-0.08 (-0.31 to 0.15)	0.47	0.05 (-0.18 to 0.27)	0.70	-0.17 (-0.4 to 0.07)	0.16	-0.14 (-0.37 to 0.09)	0.24	-0.09 (-0.19 to 0.02)	0.11	-0.08 (-0.18 to 0.03)	0.14
	Grade III	Ref	–	Ref	–	Ref	–	Ref	–	Ref	–	Ref	–
ICM	C	–	–	–	–	0.25 (-0.38 to 0.89)	0.43	0.16 (-0.49 to 0.81)	0.63	0.23 (-0.11 to 0.56)	0.18	0.18 (-0.16 to 0.51)	0.31
	B	–	–	–	–	-0.07 (-0.28 to 0.13)	0.48	-0.1 (-0.33 to 0.13)	0.39	0.06 (-0.05 to 0.16)	0.28	0.05 (-0.06 to 0.16)	0.34
	A	–	–	–	–	Ref	–	Ref	–	Ref	–	Ref	–
TE	C	–	–	–	–	0.1 (-0.44 to 0.64)	0.71	0.01 (-0.54 to 0.57)	0.96	0.17 (-0.08 to 0.42)	0.19	0.05 (-0.2 to 0.3)	0.69
	B	–	–	–	–	-0.07 (-0.23 to 0.09)	0.37	-0.03 (-0.2 to 0.13)	0.72	-0.02 (-0.11 to 0.07)	0.65	-0.05 (-0.15 to 0.04)	0.27
	A	–	–	–	–	Ref	–	Ref	–	Ref	–	Ref	–
Blastocyst score	Poor	–	–	–	–	0.15 (-0.31 to 0.6)	0.53	0.16 (-0.31 to 0.62)	0.51	**0.24 (0.02 to 0.46)**	**0.03**	0.17 (-0.06 to 0.39)	0.14
	Fair	–	–	–	–	-0.11 (-0.36 to 0.14)	0.36	-0.09 (-0.36 to 0.17)	0.49	0.05 (-0.08 to 0.17)	0.45	0.03 (-0.1 to 0.16)	0.64
	Good	–	–	–	–	-0.04 (-0.3 to 0.22)	0.77	-0.03 (-0.29 to 0.24)	0.85	0.11 (-0.03 to 0.24)	0.12	0.08 (-0.05 to 0.21)	0.23
	Top	–	–	–	–	Ref	–	Ref	–	Ref	–	Ref	–

Models were adjusted for maternal age, BMI, parity, peak estradiol level, endometrial thickness, insemination protocol (IVF or ICSI), female etiologies (tubal factor, endometriosis), order of transfer (1 or >1), mode of delivery (vaginal or Cesarean), and year of treatment. CI, confidence interval; ICM, inner cell mass; TE, trophectoderm. The bold values indicate significant at P<0.05.

We also evaluated the association between the birth weight z-score and embryo/blastocyst quality (**Table 2**). Data showed no significant association between birth weight z-score and embryo/blastocyst grades regardless of the type of transfer, except an increased birth weight z-score was noted when poor-quality blastocysts were transferred in the FBT cycles. However, the association between the birth weight z-score and blastocyst quality was no longer significant following adjustment for confounders.

The associations between the birth weight z-score and morphological parameters were also confirmed through absolute birth weight analyses (**Supplementary Table S3**). Multivariate analyses suggested that transferring a fragmented embryo may lead to a 115.4 (95% CI: 26.6–204.2)-g increase in birth weight following fresh cleavage transfer and a 168.8 (95% CI: 48.8–288.8)-g increase following fresh blastocyst transfer.

We used interaction analyses to evaluate the effects of Frozen-thawed embryo transfer (FET) on the association between morphology and birth weight. The size of association of fragmentation with the birth weight and birth weight z-score in the FBT group decreased by 142.36 (95% CI: 15.86–268.87) and 0.34 (95% CI: 0.01–0.66), respectively, in comparison with that of the BT group.

**Figure 1:**
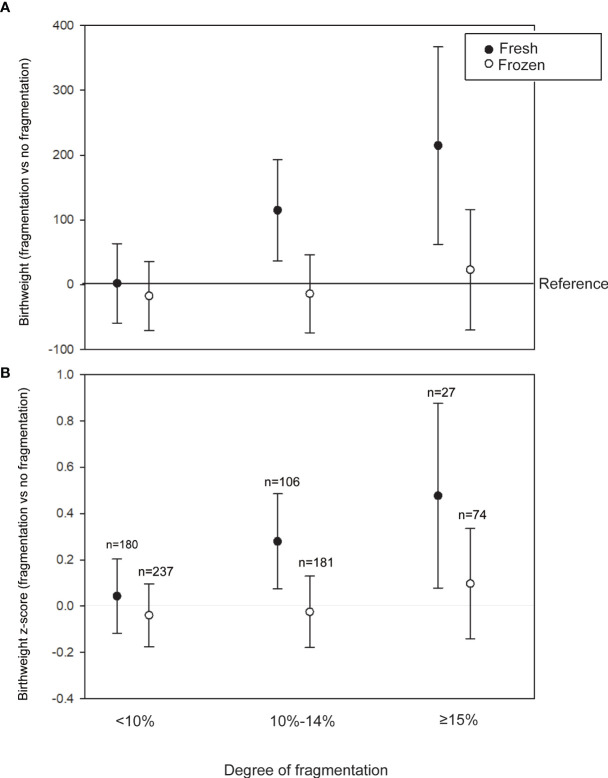
Association between the degree of fragmentation and birth weight in the fresh (cleavage and blastocyst transfer) and frozen-thawed cycles. **(A)** Adjusted regression coefficients of fragmentation degrees for birth weight using no fragmentation as a reference. **(B)** Adjusted regression coefficients of fragmentation degrees for birth weight z-score using no fragmentation as a reference. Models are adjusted for maternal age, body mass index (BMI), parity, peak estradiol level, endometrial thickness, insemination protocol *in vitro* fertilization (IVF) or intracytoplasmatic sperm injection (ICSI), female etiologies (tubal factor, endometriosis), order of transfer (1 or >1), mode of delivery (virginal or Cesarean), and year of treatment.

To examine whether birth weight increased with the severity of fragmentation, we further categorized the degree of fragmentation into four groups (no fragmentation; low fragmentation, <10%; moderate fragmentation, 10%–14%; high fragmentation, ≥15%). In fresh transfer cycles (cleavage and blastocyst stage), multivariate analyses suggested that in comparison with the no fragmentation group, the low fragmentation, moderate fragmentation, and high fragmentation groups experienced birth weight increases of 2.04 (95% CI: -59.28 to 63.36) g, 114.7 (95% CI: 36.38–193.1) g, and 214.8 (95% CI: 62.54–367.08) g, respectively. A similar trend was also observed with the birth weight z-score (**Figure 1**), suggesting a dose-dependent effect of fragmentation on birth weight. On the other hand, no dose-dependent effect of fragmentation was detected in the frozen-thawed cycles.

Since fragmentation has been associated with standardized and absolute birth weight, we also evaluate its association with other neonatal outcomes, including SGA, LGA, LBW, HBW, and preterm birth (**Table 3**). The rates of LGA appeared to be higher following the transfer of embryos with ≥10% fragmentation on day 3 (14.1% vs. 8.5%, p = 0.077 for fresh cleavage; 16.7% vs. 7.6%, p = 0.026 for fresh blastocyst), and the same was also observed for HBW (10.6% vs. 5.1%, p = 0.03 for fresh cleavage; 14.6% vs. 4.7%, p = 0.003 for fresh blastocyst). Multivariate analyses suggested that there was an approximately 2-fold increase in the odds of LGA/HBW with fragmentation in the fresh blastocyst transfer cycles.

**Table 3:** Association between day-3 fragmentation (≥10% vs. <10%) and neonatal outcomes.

	LBW		HBW		LGA		SGA		Preterm birth	
	OR (95% CI)	p	OR (95% CI)	p	OR (95% CI)	p	OR (95% CI)	p	OR (95% CI)	p
Fresh cleavage	0.81 (0.28 to 2.36)	0.70	1.91 (0.86 to 4.24)	0.11	1.59 (0.79 to 3.18)	0.19	0.69 (0.31 to 1.51)	0.35	0.5 (0.12 to 2.14)	0.35
Fresh blastocyst	0.39 (0.08 to 2.06)	0.27	**3.65 (1.33 to 10)**	**0.01**	**3 (1.2 to 7.51)**	**0.02**	N/A^a^	–	0.54 (0.09 to 3.07)	0.48
Fresh cleavage +blastocyst	0.64 (0.27 to 1.53)	0.31	**2.49 (1.36 to 4.55)**	**<0.01**	**1.99 (1.17 to 3.41)**	**0.01**	0.38 (0.18 to 0.8)	0.01	0.53 (0.19 to 1.51)	0.24
Frozen blastocyst	0.98 (0.5 to 1.89)	0.94	1.05 (0.59 to 1.87)	0.86	1.01 (0.63 to 1.62)	0.97	1.1 (0.68 to 1.79)	0.69	1.17 (0.62 to 2.22)	0.63

Models were adjusted for maternal age, BMI, parity, peak estradiol level, endometrial thickness, insemination protocol (IVF or ICSI), female etiologies (tubal factor, endometriosis), order of transfer (1 or >1), mode of delivery (vaginal or Cesarean), and year of treatment.

OR, odds ratio comparing embryos with day-3 fragmentation ≥10% vs. embryos with day-3 fragmentation <10%.

a Data were unavailable because no event was observed. LBW, low birth weight; HBW, hight birth weight; LGA, large for gestational age; SGA, small for gestational age, CI, confidence interval. The bold values indicate significant at P<0.05.

A significant modification to the association between the blastocyst morphology and birth weight by day-3 fragmentation was also observed when the interaction terms were included (**Supplementary Table S4**). Comparing the birth weight for blastocysts with grade A ICM to those with grades B and C, the size of association increased by 473.4 (95% CI: 124.04–822.76) and -70.42 (95% CI: -630.44 to 489.59), respectively, in the fragmented group compared to the no-fragmentation group. The association between the blastocyst quality and birth weight was also modified by day-3 fragmentation (**Supplementary Table S4**).

## Discussion

### Main outcomes

Our data suggested that day-3 fragmentation was associated with higher birth weight, regardless of whether cleavage-stage embryos or blastocysts were transferred in fresh cycles. Moreover, the association between blastocyst morphology and birth weight might be modified by the presence of fragmentation on day 3. In the frozen-thawed cycles, however, no association was detected between embryo morphology and birth weight. In addition, our data did not support the argument that transferring a poor-looking embryo (BC/CB/CC for blastocysts; fair or poor grade for day-3 embryos) may increase the risks of low birth weight (3, 4), as we observed no association between embryo quality and birth weight in either the fresh or frozen-thawed transfer cycles.

### Strengths and limitations

Compared with previous studies (3–11), our study may have several strengths. First, it was one of the largest studies on this topic, including singletons following both fresh and frozen-thawed transfers. Second, the data were collected in a relatively short period, minimizing the potential confounding over time (18). Finally, the study design took both the cleavage stage and blastocyst into consideration, providing more information than studies using data only related to one of these factors.

However, our study is also limited by similar methodological defects to those affecting previous studies, including the subjectivity of embryo assessment and the retrospective nature of the study. The most profound bias may lie in the fact that patients receive a poor-looking embryo only when they have no choice. Therefore, singletons following transfer of a poor-looking embryo may be more likely to be delivered by mothers with a poor prognosis. In addition, because the proportion of live births resulting from day-3 fragmented embryos remained small in the birth cohort, the association between fragmentation and birth weight should be verified in a larger cohort.

### Interpretation

Cleavage-stage embryo fragmentation has been defined as the presence of anuclear membrane-bound extracellular cytoplasmic structures and used as a marker for embryos with poor prognosis. Embryo fragmentation has been associated with reduced blastocyst formation, decreased implantation, and increased early abortion. However, its effect on birth weight is less widely known. Ebner et al. (19) demonstrated that the mean birth weights of singletons following transfer of embryos with no fragmentation, <25% fragmentation, and 25%–50% fragmentation were 3,128, 3,235, and 3,421 g, respectively. Although this early study was limited by its small sample size and the authors concluded that birth weight did not correlate with embryo quality (19), the numerical trend may indicate an association between fragmentation and birth weight.

The pathogenesis and biological significance of fragmentation in cleavage-stage embryos are not fully understood. A transmission electron microscopy study revealed that the most abundant organelles in the fragment were mitochondria (20). The determination of extracellular mitochondrial DNA content suggested that the fragment may release mitochondria into the culture medium (21). These observations may suggest a reduction in the mitochondrial number and/or a deficiency in the mitochondrial function in fragmented embryos. Alternations in the mitochondrial function during the early embryo stage may affect later development. Using a mouse model, Zander-Fox et al. (22) showed that disrupting the mitochondrial oxidative phosphorylation process during the cleavage-stage embryo culture might reduce the birth weight and impair the metabolic phenotypes of the offspring (22). In humans, mitochondrial DNA depletion was found in both SGA and LGA neonates (23). Together with the existing evidence, the present study suggests that further research is warranted to investigate the putative links between early embryo morphology, mitochondrial dysfunction, and later life development.

Among the studies investigating the association between embryo quality and birth weight, Zhang et al. (4) suggested an association between poor blastocyst morphology and low birth weight, whereas Huang et al. (3) demonstrated the greater risk of low birth weight following the transfer of poor-quality cleavage-stage embryos in the same population. The inconsistency between their conclusions and ours may lie in the subjectivity of the embryo assessment and the different embryo selection criteria. However, the authors of these studies also reported a relatively high birth weight z-score and an LGA rate of up to 37.5% in singletons from top-quality embryos. In contrast, our data demonstrated a population with far lower birth weight z-scores and LGA rates using the same reference population (17). This suggests that significant differences in study cohorts or regions may contribute to the heterogeneity of such studies. For instance, the distribution of socioeconomic factors (24) and maternal lifestyles (25) may vary among cohorts or regions, leading to varying baseline birth weight z-scores. On the other hand, the lack of association between embryo quality and birth weight observed in our study was supported by several other studies (5–7, 10).

Several studies have associated singleton birth weight with specific morphological features rather than the overall embryo score, yielding conflicting results (9–11). Licciardi et al. (9) reported that a higher ICM grade was associated with a higher birth weight, but the TE grade did not relate to the weight. Controversially, Bakkensen et al. (10) reported a negative association between ICM grades and birth weight. In a more recent study, advanced TE grade, rather than ICM, was found to be associated with increased birth weight (11). A possible reason for this discrepancy is that the limited choices in terms of ICM and TE grades, in combination with subjectivity, may limit the discriminatory power of the grading system. The majority of blastocysts (60%–70%) transferred were graded “B” for either TE or ICM. While it is easier to discriminate between TE and ICM with extremely high or low cell numbers, TE or ICM with intermediate-high or intermediate-low cell numbers may be classified as higher or lower grade according to different embryologists. A misclassification of the intermediate grade “B” may skew the estimation for both the higher and lower grades. When a more quantitative grading method was introduced, Ebner et al. (26) reported no association between birth weight and either TE or ICM. However, this study only included 99 live births and needs to be confirmed in larger cohorts.

The association between blastocyst morphology and developmental potential might also be affected by their cleavage-stage origin. Recent studies have suggested that the developmental potential of blastocysts might differ according to their different morphological features in the cleavage stage, even though a similar blastocyst grade may be given (12–14). In advanced good-quality blastocysts, the degree of fragmentation on day 3 may have a negative impact on the implantation potential (12). The observations of Hardy et al. (27) have revealed an interrelation between fragmentation and blastocyst morphology. Embryos with minimal or moderate levels of fragmentation (5%–25%) in the cleavage stage had a reduced TE cell number but maintained a steady cell number in ICM in the blastocyst stage. However, the levels of apoptosis in the ICM were high in blastocysts from embryos without fragmentation but lower in blastocysts from fragmented embryos (27). Apoptosis in ICM may play a crucial role in eliminating defective cells and regulating cell numbers (28). Therefore, the observations of Hardy et al. (27) may suggest that imbalanced ICM/TE development and/or dysregulation of ICM growth occurs in blastocysts following fragmentation, even though the morphology is similar to that of a blastocyst from embryos without fragmentation. ICM dysregulation is postulated to be associated with fetal overgrowth in animals (29). A decreased ICM/TE ratio may indicate an imbalance in placental development and explain some of the features of large calf syndrome (29). Echoing earlier observations, our interaction analysis suggested that an association between a lower number of ICM cells and large babies may also exist in humans, but only when fragmentation is present in the cleavage stage.

Our study failed to associate any morphological features with birth weight in frozen-thawed cycles, and the data suggested a significant modification of frozen-thawed on our estimate. This was not the first time that data have suggested that technologies may modify the association between embryo morphology and neonatal outcomes. Lieberman et al. (8) showed that the cell number on day 3 is positively associated with birth weight but only without assisted hatching. The authors hypothesized that creating an artificial hole in the zona pellucida by assisted hatching may alter the embryonic metabolism and affect subsequent development. Similarly, vitrification may lead to a wide range of alternations in the cellular structure, including damage to the mitochondria and cytoskeleton (30, 31) and has been associated with alternated neonatal outcomes (32). The effects of day-3 fragmentation on birth weight might be overwhelmed by the changes made due to vitrification, as the impacts of the latter equally affect embryos with and without day-3 fragmentation. On the other hand, the observation might be explained by the hypothesis that vitrification sets an additional selection barrier for embryos to screen for the fitness of surviving a freeze-thaw cycle (33). If blastocysts with day-3 fragmentation were less likely to pass the barrier and achieve a live birth than their counterparts without day-3 fragmentation, a potential bias may affect the association between day-3 fragmentation and birth weight outcomes. However, when we checked the proportion of embryos with day-3 fragmentation at each step of the treatment (**Supplementary Table S5**), the proportion of blastocysts with day-3 fragmentation among all blastocysts transferred in FBT cycles was found to be similar to the proportion of blastocysts with day-3 fragmentation among blastocysts that achieved a live birth (**Supplementary Table S5**). Therefore, the later hypothesis is not supported by our data.

## Conclusions

In conclusion, our data suggested that fragmentation in the cleavage stage is associated with increased birth weight following either cleavage- or blastocyst-stage transfers in fresh cycles but not in frozen-thawed cycles. Transferring day-3-fragmented embryos appeared to be safe for offspring, as infants with a higher birth weight may show positive long-term health, development, and educational outcomes (34). However, concerns for LGA infants remain because the risks of adult-onset diseases may also increase in those with an extremely high birth weight (35).

## Data availability statement

The original contributions presented in the study are included in the article/**Supplementary Materials**. Further inquiries can be directed to the corresponding author.

## Ethics statement

This study was reviewed and approved by Ethical Committee of the Xiamen University Affiliated Chenggong Hospital. The patients/participants provided their written informed consent to participate in this study.

## Author contributions

JC, LL and JR contribute to conception and design. JHC, ZL and XJ contribute to the acquisition of data. JLC and LL contribute to the analysis and interpretation of data. All authors contributed to drafting the article or revising it critically for important intellectual content. All authors read and approved the final manuscript.

## Funding

This work was supported by the National Natural Science Foundation of China [grant number 22176159]; the Xiamen medical advantage subspecialty construction project [grant number 2018296]; the Special Fund for Clinical and Scientific Research of Chinese Medical Association [grant number 18010360765]; Xiamen Science and Technology Benefit Plan [grant number 3502Z20194053].

## Acknowledgments

We thank Xinli Wang for her assistance in the data processing.

## Conflict of interest

The authors declare that the research was conducted in the absence of any commercial or financial relationships that could be construed as a potential conflict of interest.

## Publisher’s note

All claims expressed in this article are solely those of the authors and do not necessarily represent those of their affiliated organizations, or those of the publisher, the editors and the reviewers. Any product that may be evaluated in this article, or claim that may be made by its manufacturer, is not guaranteed or endorsed by the publisher.

## References

[1] Gardner DK Lane M Stevens J Schlenker T Schoolcraft WB. Blastocyst score affects implantation and pregnancy outcome: Towards a single blastocyst transfer. Fertil Steril (2000) 73(6):1155–8. doi: 10.1016/s0015-0282(00)00518-5 10856474

[2] Alpha Scientists in Reproductive, M., and Embryology, E.S.I.G.o. The Istanbul consensus workshop on embryo assessment: Proceedings of an expert meeting. Hum Reprod (2011) 26(6):1270–83. doi: 10.1093/humrep/der037 21502182

[3] Huang J Tao Y Zhang J Yang X Wu J Kuang Y. Poor embryo quality is associated with a higher risk of low birthweight in vitrified-warmed single embryo transfer cycles. Front Physiol (2020) 11:415. doi: 10.3389/fphys.2020.00415 32499716 PMC7243353

[4] Zhang J Huang J Liu H Wang B Yang X Shen X. The impact of embryo quality on singleton birthweight in vitrified-thawed single blastocyst transfer cycles. Hum Reprod (2020) 35(2):308–16. doi: 10.1093/humrep/dez287 32020183

[5] Oron G Son WY Buckett W Tulandi T Holzer H. The association between embryo quality and perinatal outcome of singletons born after single embryo transfers: A pilot study. Hum Reprod (2014) 29(7):1444–51. doi: 10.1093/humrep/deu079 24812313

[6] Zhu J Lian Y Li M Chen L Liu P Qiao J. Does IVF cleavage stage embryo quality affect pregnancy complications and neonatal outcomes in singleton gestations after double embryo transfers? J Assist Reprod Genet (2014) 31(12):1635–41. doi: 10.1007/s10815-014-0351-8 PMC4250469 25326318

[7] Bouillon C Celton N Kassem S Frapsauce C Guerif F. Obstetric and perinatal outcomes of singletons after single blastocyst transfer: Is there any difference according to blastocyst morphology? Reprod BioMed Online (2017) 35(2):197–207. doi: 10.1016/j.rbmo.2017.04.009 28601377

[8] Lieberman E Ginsburg ES Racowsky C. Rate of cell division and weight of neonates following IVF. Reprod BioMed Online (2006) 12(3):315–21. doi: 10.1016/s1472-6483(10)61003-6 16569319

[9] Licciardi F McCaffrey C Oh C Schmidt-Sarosi C McCulloh DH. Birth weight is associated with inner cell mass grade of blastocysts. Fertil Steril (2015) 103(2):382–7.e382. doi: 10.1016/j.fertnstert.2014.10.039 25497449

[10] Bakkensen JB Brady P Carusi D Romanski P Thomas AM Racowsky C. Association between blastocyst morphology and pregnancy and perinatal outcomes following fresh and cryopreserved embryo transfer. J Assist Reprod Genet (2019) 36(11):2315–24. doi: 10.1007/s10815-019-01580-0 PMC6885471 31512049

[11] Xie Q Du T Zhao M Gao C Lyu Q Suo L. Advanced trophectoderm quality increases the risk of a large for gestational age baby in single frozen-thawed blastocyst transfer cycles. Hum Reprod (2021) 36(8):2111-20. doi: 10.1093/humrep/deab088 33956949

[12] della Ragione T Verheyen G Papanikolaou EG Van Landuyt L Devroey P Van Steirteghem A. Developmental stage on day-5 and fragmentation rate on day-3 can influence the implantation potential of top-quality blastocysts in IVF cycles with single embryo transfer. Reprod Biol Endocrinol (2007) 5:2. doi: 10.1186/1477-7827-5-2 17257401 PMC1796880

[13] Wu J Zhang J Kuang Y Chen Q Wang Y. The effect of day 3 cell number on pregnancy outcomes in vitrified-thawed single blastocyst transfer cycles. Hum Reprod (2020) 35(11):2478–87. doi: 10.1093/humrep/deaa209 32944763

[14] Zhao H Liu H Li M Wu K. Clinical outcomes following frozen-thawed blastocyst transfers with blastocysts derived from different cell numbers on day 3: A retrospective cohort study. J Assist Reprod Genet (2020) 37(3):641–8. doi: 10.1007/s10815-019-01664-x PMC7125268 31902101

[15] Skiadas CC Jackson KV Racowsky C. Early compaction on day 3 may be associated with increased implantation potential. Fertil Steril (2006) 86(5):1386–91. doi: 10.1016/j.fertnstert.2006.03.051 16978618

[16] Cai J Liu L Zhang J Qiu H Jiang X Li P. Low body mass index compromises live birth rate in fresh transfer *in vitro* fertilization cycles: A retrospective study in a Chinese population. Fertil Steril (2017) 107(2):422–9.e422. doi: 10.1016/j.fertnstert.2016.10.029 27887711

[17] Dai L Deng C Li Y Zhu J Mu Y Deng Y. Birth weight reference percentiles for Chinese. PLoS One (2014) 9(8):e104779. doi: 10.1371/journal.pone.0104779 25127131 PMC4134219

[18] Castillo CM Horne G Fitzgerald CT Johnstone ED Brison DR Roberts SA. The impact of IVF on birthweight from 1991 to 2015: A cross-sectional study. Hum Reprod (2019) 34(5):920–31. doi: 10.1093/humrep/dez025 30868153

[19] Ebner T Yaman C Moser M Sommergruber M Polz W Tews G. Embryo fragmentation *in vitro* and its impact on treatment and pregnancy outcome. Fertil Steril (2001) 76(2):281–5. doi: 10.1016/s0015-0282(01)01904-5 11476773

[20] Halvaei I Khalili MA Esfandiari N Safari S Talebi AR Miglietta S. Ultrastructure of cytoplasmic fragments in human cleavage stage embryos. J Assist Reprod Genet (2016) 33(12):1677–84. doi: 10.1007/s10815-016-0806-1 PMC5171886 27614632

[21] Stigliani S Anserini P Venturini PL Scaruffi P. Mitochondrial DNA content in embryo culture medium is significantly associated with human embryo fragmentation. Hum Reprod (2013) 28(10):2652–60. doi: 10.1093/humrep/det314 23887072

[22] Zander-Fox DL Fullston T McPherson NO Sandeman L Kang WX Good SB. Reduction of mitochondrial function by FCCP during mouse cleavage stage embryo culture reduces birth weight and impairs the metabolic health of offspring. Biol Reprod (2015) 92(5):124. doi: 10.1095/biolreprod.114.123489 25715796

[23] Gemma C Sookoian S Alvarinas J Garcia SI Quintana L Kanevsky D. Mitochondrial DNA depletion in small- and large-for-gestational-age newborns. Obes (Silver Spring) (2006) 14(12):2193–9. doi: 10.1038/oby.2006.257 17189546

[24] He JR Li WD Lu MS Guo Y Chan FF Lu JH. Birth weight changes in a major city under rapid socioeconomic transition in China. Sci Rep (2017) 7(1)1031. doi: 10.1038/s41598-017-01068-w 28432291 PMC5430650

[25] Tang L Pan XF Lee AH Binns CW Yang CX Sun X. Maternal lifestyle and nutritional status in relation to pregnancy and infant health outcomes in Western China: protocol for a prospective cohort study. BMJ Open (2017) 7(6):e014874. doi: 10.1136/bmjopen-2016-014874 PMC5541627 28630084

[26] Ebner T Tritscher K Mayer RB Oppelt P Duba HC Maurer M. Quantitative and qualitative trophectoderm grading allows for prediction of live birth and gender. J Assist Reprod Genet (2016) 33(1):49–57. doi: 10.1007/s10815-015-0609-9 26572782 PMC4717145

[27] Hardy K Stark J Winston RM. Maintenance of the inner cell mass in human blastocysts from fragmented embryos. Biol Reprod (2003) 68(4):1165–9. doi: 10.1095/biolreprod.102.010090 12606492

[28] Pisko J Spirkova A Cikos S Olexikova L Kovarikova V Sefcikova Z. Apoptotic cells in mouse blastocysts are eliminated by neighbouring blastomeres. Sci Rep (2021) 11(1):9228. doi: 10.1038/s41598-021-88752-0 33927296 PMC8085119

[29] Leese HJ Donnay I Thompson JG. Human assisted conception: A cautionary tale. lessons from domestic animals. Hum Reprod (1998) 13)Suppl 4):184–202. doi: 10.1093/humrep/13.suppl_4.184 10091069

[30] Chatzimeletiou K Morrison EE Panagiotidis Y Vanderzwalmen P Prapas N Prapas Y. Cytoskeletal analysis of human blastocysts by confocal laser scanning microscopy following vitrification. Hum Reprod (2012) 27(1):106–13. doi: 10.1093/humrep/der344 22028018

[31] Gao Z Yao G Zhang H Liu H Yang Z Liu C. Resveratrol protects the mitochondria from vitrification injury in mouse 2-cell embryos. Cryobiology (2020) 95:123–9. doi: 10.1016/j.cryobiol.2020.05.007 32464144

[32] Zaat T Zagers M Mol F Goddijn M van Wely M Mastenbroek S. Fresh versus frozen embryo transfers in assisted reproduction. Cochrane Database Syst Rev (2021) 2:CD011184. doi: 10.1002/14651858.CD011184.pub3 33539543 PMC8095009

[33] Hanevik HI Hessen DO. IVF and human evolution. Hum Reprod Update (2022) 28(4):457–79. doi: 10.1093/humupd/dmac014 35355060

[34] Zeng P Zhou X. Causal association between birth weight and adult diseases: Evidence from a mendelian randomization analysis. Front Genet (2019) 10:618. doi: 10.3389/fgene.2019.00618 31354785 PMC6635582

[35] Knop MR Geng TT Gorny AW Ding R Li C Ley SH. Birth weight and risk of type 2 diabetes mellitus, cardiovascular disease, and hypertension in adults: A meta-analysis of 7 646 267 participants from 135 studies. J Am Heart Assoc (2018) 7(23):e008870. doi: 10.1161/JAHA.118.008870 30486715 PMC6405546

