# Supplementary material for: Day-3-embryo fragmentation is associated with singleton birth weight following fresh single blastocyst transfer: A retrospective study
Source: Front Endocrinol (Lausanne). 2022 Sep 23;13:919283. doi: 10.3389/fendo.2022.919283 (PMC9538176; doi:10.3389/fendo.2022.919283)
Supplement: Supplementary file 1 [file Table_1.docx]

Table S1 Patients characteristics and neonatal outcomes in frozen-thawed cleavage stage transfer cycles resulting in singletons

| Variable |  |  |
| --- | --- | --- |
| N |  | 127 |
| Maternal age, year |  | 31 [28-34] |
| Maternal BMI, kg/cm2 | | 21 [19.6-22.6] |
| Maternal etiology | Tubal | 12 (9.4) |
|  | Endometriosis | 75 (59.1) |
| Insemination | IVF | 93 (73.2) |
|  | ICSI | 34 (26.8) |
| ET order | 1 | 5 (3.9) |
|  | >1 | 122 (96.1) |
| Parity | 0 | 95 (74.8) |
|  | ≧1 | 32 (25.2) |
| Endometrial thickness, mm | | 9 [8-10.4] |
| Peak estradiol level, pg/L | | 265 [187-392] |
| Year of treatment | 2016 | 15 (11.8) |
|  | 2017 | 28 (22) |
|  | 2018 | 43 (33.9) |
|  | 2019 | 41 (32.3) |
| Embryo parameters |  |  |
| Early cleavage |  | 69 (54.3) |
| Day 3 fragmentation≧10% | | 14 (11) |
| Day 3 cleavage | 8 cells | 65 (51.2) |
|  | < 8 cells | 37 (29.1) |
|  | > 8cells | 25 (19.7) |
| Day 3 asymmetry |  | 34 (26.8) |
| Blastocyst ICM | A | - |
|  | B | - |
|  | C | - |
| Blastocyst TE | A | - |
|  | B | - |
|  | C | - |
| Cleavage score | Grade III | 23 (18.1) |
|  | Grade II | 101 (79.5) |
|  | Grade I | 3 (2.4) |
| Blastocyst score | Top |  |
|  | Good |  |
|  | Fair |  |
|  | Poor |  |
| Outcomes |  |  |
| Mode of delivery | Virginal | 37 (29.1) |
|  | Cesarean | 90 (70.9) |
| Offspring gender | Female | 61 (48) |
|  | Male | 66 (52) |
| Birthweight, g |  | 3250 [2950-3530] |
| Z-score |  | 0.06 [-0.65 to 0.55] |
| Gestational age, week | | 39 [38-40] |
| Preterm birth |  | 10 (7.9) |
| LBW, <2500g |  | 12 (9.4) |
| HBW, >4000g |  | 4 (3.1) |
| LGA |  | 12 (9.4) |
| SGA |  | 12 (9.4) |
