# Supplementary material for: Day-3-embryo fragmentation is associated with singleton birth weight following fresh single blastocyst transfer: A retrospective study
Source: Front Endocrinol (Lausanne). 2022 Sep 23;13:919283. doi: 10.3389/fendo.2022.919283 (PMC9538176; doi:10.3389/fendo.2022.919283)
Supplement: Supplementary file 2 [file Table_2.docx]

Table S2 Association of embryo morphological features with z-score and birthweight in frozen-thawed cycles resulting in singletons

|  |  |  |  |  |  |  |  |  |  |
| --- | --- | --- | --- | --- | --- | --- | --- | --- | --- |
|  |  | z-score | | | | birthweight | | | |
|  |  | unadjusted | | adjusted | | unadjusted | | adjusted | |
|  |  | Coefficient (95% CI) | P | Coefficient (95% CI) | P | Coefficient (95% CI) | P | Coefficient (95% CI) | P |
| Early cleavage | yes | 0.02 (-0.35 to 0.39) | 0.918 | 0.08 (-0.3 to 0.45) | 0.687 | 141.4 (-37.61 to 320.4) | 0.122 | 36.58 (-117.81 to 190.96) | 0.642 |
|  | no | Ref | - | Ref | - | Ref | - | Ref | - |
| Fragmentation | ≧10% | -0.35 (-0.95 to 0.24) | 0.24 | -0.43 (-1.03 to 0.18) | 0.164 | -102.84 (-389.66 to 183.99) | 0.482 | -217.59 (-458.48 to 23.31) | 0.077 |
|  | <10% | Ref | - | Ref | - | Ref | - | Ref | - |
| Cell number | >8 cells | -0.22 (-0.71 to 0.27) | 0.373 | -0.15 (-0.65 to 0.35) | 0.555 | -154.59 (-390.81 to 81.64) | 0.2 | -90.06 (-290.88 to 110.76) | 0.379 |
|  | <8 cells | -0.24 (-0.67 to 0.19) | 0.267 | -0.25 (-0.69 to 0.2) | 0.273 | -144.74 (-351.45 to 61.98) | 0.17 | -80.64 (-259.22 to 97.94) | 0.376 |
|  | 8 cells | Ref | - | Ref | - | Ref | - | Ref | - |
| Symmentry | uneven | -0.14 (-0.56 to 0.28) | 0.518 | -0.12 (-0.55 to 0.31) | 0.581 | -76.58 (-279.42 to 126.25) | 0.459 | -29.9 (-203.68 to 143.88) | 0.736 |
|  | even | Ref | - | Ref | - | Ref | - | Ref | - |
| Cleavage score | Grade I | 0.8 (-0.48 to 2.07) | 0.221 | 0.99 (-0.3 to 2.28) | 0.133 | 388.7 (-229.48 to 1006.87) | 0.218 | 386.53 (-139.17 to 912.23) | 0.15 |
|  | Grade II | 0.28 (-0.2 to 0.76) | 0.257 | 0.35 (-0.15 to 0.85) | 0.17 | -18.48 (-251.15 to 214.19) | 0.876 | 154.42 (-48.43 to 357.26) | 0.136 |
|  | Grade III | Ref | - | Ref | - | Ref | - | Ref | - |

Models were adjusted for maternal age, BMI, parity, peak estradiol level, endometrial thickness, insemination protocol (IVF or ICSI), female etiologies (tubal factor, endometriosis), order of transfer (1 or >1), mode of delivery (virginal or cesarean) and year of treatment. Analyses for birthweight were also adjusted for gestational age and gender.
