# Supplementary material for: Day-3-embryo fragmentation is associated with singleton birth weight following fresh single blastocyst transfer: A retrospective study
Source: Front Endocrinol (Lausanne). 2022 Sep 23;13:919283. doi: 10.3389/fendo.2022.919283 (PMC9538176; doi:10.3389/fendo.2022.919283)
Supplement: Supplementary file 3 [file Table_3.docx]

Table S3 Association between embryo morphological features and birthweight

| Variable | Category | Fresh cleavage transfer | | | | Fresh blastocyst transfer | | | | Frozen blastocyst transfer | | | |
| --- | --- | --- | --- | --- | --- | --- | --- | --- | --- | --- | --- | --- | --- |
|  |  | unadjusted | | adjusted | | unadjusted | | adjusted | | unadjusted | | adjusted | |
|  |  | coefficient（95%CI） | P | coefficient（95%CI） | P | coefficient（95%CI） | P | coefficient（95%CI） | P | coefficient（95%CI） | P | coefficient（95%CI） | P |
| Early cleavage | yes | -1.98 (-60.3 to 56.33) | 0.947 | 9.09 (-41.3 to 59.5) | 0.724 | 42.3 (-32.9 to 117.5) | 0.271 | 36.2 (-29.4 to 101.9) | 0.28 | 13.3 (-27.9 to 54.5) | 0.527 | 10.8 (-25.1 to 46.7) | 0.555 |
|  | no | Ref | - | Ref | - | Ref | - | Ref | - | Ref | - | Ref | - |
| Fragmentation | ≧10% | **159.8 (56.9 to 262.5)** | **0.002** | **115.4 (26.6 to 204.2)** | **0.011** | 128 (-10.8 to 266.8) | 0.071 | **168.8 (48.8 to 288.8)** | **0.006** | 13.21 (-50.1 to 76.5) | 0.682 | 7.47 (-46.4 to 61.3) | 0.786 |
|  | <10% | Ref | - | Ref | - | Ref | - | Ref | - | Ref | - | Ref | - |
| Cleavage | >8 cells | **98.2 (7.1 to 189.3)** | **0.035** | 57.77 (-22.21 to 137.75) | 0.157 | -37.92 (-120.69 to 44.85) | 0.369 | -11.6 (-84.25 to 61.05) | 0.754 | 31.92 (-15.8 to 79.65) | 0.19 | 23.56 (-17.19 to 64.31) | 0.257 |
|  | <8 cells | 29.25 (-54.96 to 113.46) | 0.496 | -34.37 (-111.39 to 42.65) | 0.382 | 37.04 (-70.35 to 144.42) | 0.499 | 45.89 (-45.43 to 137.21) | 0.325 | **56.52 (7.18 to 105.85)** | **0.025** | 33.19 (-9.47 to 75.86) | 0.127 |
|  | 8 cells | Ref | - | Ref | - | Ref | - | Ref | - | Ref | - | Ref | - |
| Symmetry | uneven | 3.41 (-74.5 to 81.32) | 0.932 | -34.26 (-104.25 to 35.74) | 0.337 | 5.74 (-86.88 to 98.36) | 0.903 | 23.6 (-58.22 to 105.42) | 0.572 | 40 (-7.29 to 87.29) | 0.097 | **44.73 (4.49 to 84.96)** | **0.029** |
|  | even | Ref | - | Ref | - | Ref | - | Ref | - | Ref | - | Ref | - |
| Cleavage score | Grade I | -64.1 (-193.61 to 65.42) | 0.332 | 13.03 (-102.08 to 128.14) | 0.824 | -103.32 (-292.44 to 85.8) | 0.284 | -80.03 (-239.69 to 79.63) | 0.326 | -49.77 (-161.91 to 62.36) | 0.384 | -63.24 (-156.65 to 30.17) | 0.185 |
|  | Grade II | -69.04 (-169.39 to 31.31) | 0.178 | 15.03 (-72.99 to 103.05) | 0.738 | -33.2 (-136.57 to 70.17) | 0.529 | -62.72 (-150.07 to 24.63) | 0.159 | -38.66 (-86.8 to 9.47) | 0.115 | -35.6 (-75.87 to 4.67) | 0.083 |
|  | Grade III | Ref | - | Ref | - | Ref | - | Ref | - | Ref | - | Ref | - |
| ICM | C | - | - | - | - | 7.74 (-274.33 to 289.81) | 0.957 | 99.98 (-146.93 to 346.88) | 0.427 | 72.25 (-82.17 to 226.66) | 0.359 | 50.42 (-80.66 to 181.5) | 0.451 |
|  | B | - | - | - | - | -35.08 (-125.97 to 55.82) | 0.449 | -11.36 (-98.42 to 75.7) | 0.798 | 38.94 (-8.64 to 86.51) | 0.109 | 9 (-33.19 to 51.18) | 0.676 |
|  | A | - | - | - | - | Ref | - | Ref | - | Ref | - | Ref | - |
| TE | C | - | - | - | - | 53.57 (-185.96 to 293.1) | 0.661 | 24.94 (-186.03 to 235.91) | 0.817 | 78.86 (-36.47 to 194.19) | 0.18 | 8.7 (-89.88 to 107.29) | 0.863 |
|  | B | - | - | - | - | -43.31 (-112.57 to 25.96) | 0.22 | -5.88 (-68.67 to 56.92) | 0.854 | -8.6 (-49.22 to 32.03) | 0.678 | -22.21 (-58.52 to 14.1) | 0.231 |
|  | A | - | - | - | - | Ref | - | Ref | - | Ref | - | Ref | - |
| Blastocyst score | Poor | - | - | - | - | 17.4 (-185.5 to 220.4) | 0.866 | 93.17 (-83.62 to 269.96) | 0.302 | 93.53 (-9.44 to 196.5) | 0.075 | 45.34 (-41.01 to 131.68) | 0.303 |
|  | Fair | - | - | - | - | -64.32 (-174.56 to 45.92) | 0.253 | -15.21 (-115 to 84.57) | 0.765 | 26.15 (-32.17 to 84.47) | 0.379 | -1.07 (-50.43 to 48.29) | 0.966 |
|  | Good | - | - | - | - | -28.78 (-145.62 to 88.07) | 0.629 | -11.31 (-112.96 to 90.33) | 0.827 | 37.15 (-25.04 to 99.35) | 0.242 | 19.06 (-32.39 to 70.5) | 0.468 |
|  | Top | - | - | - | - | Ref | - | Ref | - | Ref | - | Ref | - |

Models were adjusted for maternal age, BMI, parity, peak estradiol level, endometrial thickness, insemination protocol (IVF or ICSI), female etiologies (tubal factor, endometriosis), order of transfer (1 or >1), mode of delivery (virginal or cesarean), year of treatment, gestational age and gender.
