# Supplementary material for: Day-3-embryo fragmentation is associated with singleton birth weight following fresh single blastocyst transfer: A retrospective study
Source: Front Endocrinol (Lausanne). 2022 Sep 23;13:919283. doi: 10.3389/fendo.2022.919283 (PMC9538176; doi:10.3389/fendo.2022.919283)
Supplement: Supplementary file 4 [file Table_4.docx]

Table S4 Interaction between blastocyst morphology and day 3 fragmentation on birthweight and z-score

| **Birthweight** |  | Fragmentation vs no fragmentation | |
| --- | --- | --- | --- |
| Features | Category | BT | FET |
| ICM | C vs A | -70.42 (-630.44 to 489.59) | 212.44 (-103.53 to 528.41) |
|  | B vs A | **473.4 (124.04 to 822.76)** | 33.13 (-150.19 to 216.46) |
| TE | C vs A | 14.58 (-612.77 to 641.94) | -34.34 (-262.34 to 193.67) |
|  | B vs A | -164.69 (-417.74 to 88.36) | 89.53 (-41.64 to 220.71) |
| Overall quality | Poor vs Top | 209.32 (-286.22 to 704.87) | 99.26 (-202.96 to 401.48) |
|  | Fair vs Top | 388.68 (-3.65 to 781.01) | 119.35 (-146.5 to 385.2) |
|  | Good vs Top | **668.57 (246.31 to 1090.83)** | 55.06 (-226.08 to 336.2) |
| **Z-score** |  | Fragmentation vs no fragmentation | |
| Features | Category | BT | FET |
| ICM | C vs A | -0.25 (-1.73 to 1.23) | 0.65 (-0.16 to 1.46) |
|  | B vs A | **1.17 (0.24 to 2.09)** | 0.11 (-0.36 to 0.58) |
| TE | C vs A | -0.07 (-1.72 to 1.58) | -0.05 (-0.64 to 0.53) |
|  | B vs A | -0.47 (-1.14 to 0.2) | 0.27 (-0.07 to 0.61) |
| Overall quality | Poor vs Top | 0.44 (-0.87 to 1.75) | 0.41 (-0.36 to 1.19) |
|  | Fair vs Top | 0.93 (-0.11 to 1.96) | 0.43 (-0.25 to 1.12) |
|  | Good vs Top | **1.67 (0.56 to 2.79)** | 0.27 (-0.45 to 0.99) |

Models were adjusted for maternal age, BMI, parity, peak estradiol level, endometrial thickness, insemination protocol (IVF or ICSI), female etiologies (tubal factor, endometriosis), order of transfer (1 or >1), mode of delivery (virginal or cesarean) and year of treatment. Analyses for birthweight were also adjusted for gestational age and gender.
