# Supplementary material for: Day-3-embryo fragmentation is associated with singleton birth weight following fresh single blastocyst transfer: A retrospective study
Source: Front Endocrinol (Lausanne). 2022 Sep 23;13:919283. doi: 10.3389/fendo.2022.919283 (PMC9538176; doi:10.3389/fendo.2022.919283)
Supplement: Supplementary file 5 [file Table_5.docx]

Table S5 Proportion of embryos with day 3 fragmentation at each step of the treatment

|  | Embryos for blastocyst culture | All blastocysts | Poor blastocysts | Single fresh BT ^a^ | single FBT ^a^ | live birth following single fresh BT | live birth following single FBT |
| --- | --- | --- | --- | --- | --- | --- | --- |
| Overall | 60461 | 34319 | 7888 | 1451 | 4936 | 787 | 2315 ^b^ |
| Embryos with day 3 fragmentation≧10% | 12204 | 3853 | 1726 | 94 | 590 | 48 | 246^b^ |
| Percentage of embryos with day 3 fragmentation (95% CI) | 20.18(19.86 to 20.5) | 11.23(10.89 to 11.56) | 21.88(20.97 to 22.79) | 6.48(5.21 to 7.74) | 11.95(11.05 to 12.86) | 6.1(4.43 to 7.77) | 10.63(9.37 to 11.88) |

BT, blastocyst transfer; FBT, frozen-thawed blastocyst transfer; CI, confidence interval.

^a^ Exclusion criteria were uterine abnormalities, PCOS, day3 compaction, and glucose intolerance.

^b^ Including live births that did not the inclusion criteria of the study, including monozygotic twin and gestational complications.
