# Supplementary material for: Day-3-embryo fragmentation is associated with singleton birth weight following fresh single blastocyst transfer: A retrospective study
Source: Front Endocrinol (Lausanne). 2022 Sep 23;13:919283. doi: 10.3389/fendo.2022.919283 (PMC9538176; doi:10.3389/fendo.2022.919283)
Supplement: Supplementary file 6 [file DataSheet_1.pdf]

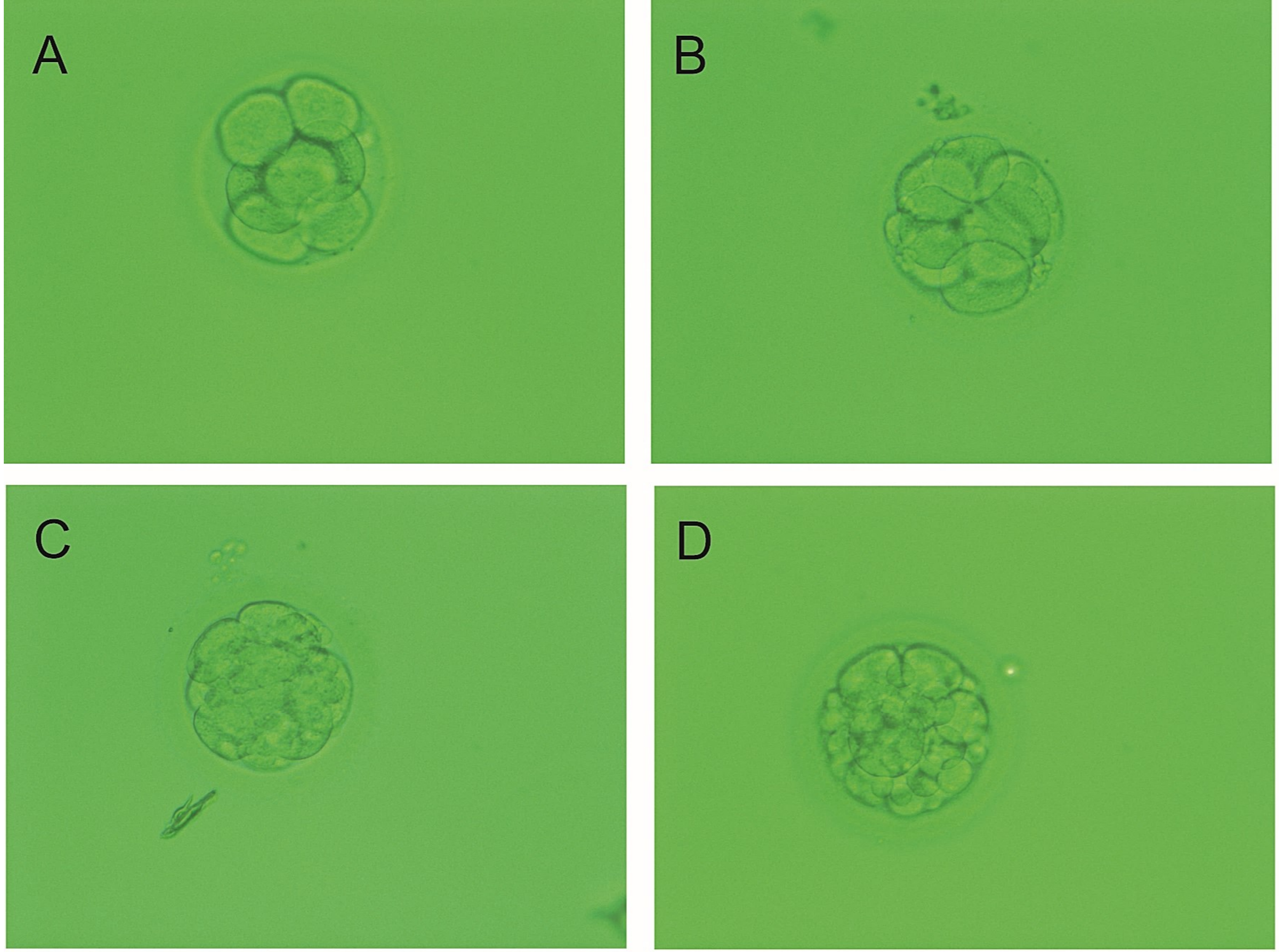

Figure S1 Representative images for day 3 fragmentation. (A) A day 3 embryo without fragmentation; (B) A day 3 embryo with <10% fragmentation; (C) A day 3 embryo with 10%-15% fragmentation; (D) A day 3 embryo with >=20% fragmentation.
